# Advancing and integrating climate and health policy in the United States: Insights from national policy stakeholders

**DOI:** 10.1016/j.joclim.2025.100485

**Published:** 2025-09-04

**Authors:** Julia Fine, Joshua Ettinger, John Kotcher, Matto Mildenberger, Anthony Leiserowitz, Edward Maibach

**Affiliations:** a George Mason University Center for Climate Change Communication, 4085/4087 University Dr, Fairfax, VA 22030, United States; b Department of Political Science, University of California Santa Barbara, Santa Barbara, CA 93106-9420, United States; c Yale Program on Climate Change Communication, Yale School of the Environment, 195 Prospect Street, New Haven, CT 06511, United States

**Keywords:** Climate change, Health, Policymaking, United States, Advocacy, Interviews

## Abstract

**Introduction:**

Many experts have called for integrating climate policy with health policy. We investigated U.S. federal policy stakeholders’ views on these goals and strategies for achieving them.

**Materials and methods:**

We conducted 65 semi-structured interviews from January 2024 to April 2024 with stakeholders working on climate policy, health policy, the climate-health intersection, and related areas. We performed a qualitative content analysis of these interviews.

**Results:**

Most stakeholders perceived that federal climate policy and health policy were mostly separate, but were becoming more integrated. They believed further integration could increase support for climate policy and maximize its health benefits. Barriers included lack of funding; competing priorities; conservative opposition; low public awareness; lack of data; and silos in federal agencies and professional communities. Opportunities included growing awareness and policy support; new funding sources, data, and technologies; the president’s ability to take executive actions; policy windows in diverse sectors, including agriculture, transportation, and housing; and potential healthcare cost savings. Proposed strategies included enhancing communication, education, and research; strengthening intra- and interagency initiatives; participatory policymaking; mobilizing existing funding; focusing first on politically feasible policies; and persistent advocacy.

**Discussion:**

These results extend previous observations of separations between climate policy and health policy and suggest ways to address these separations.

**Conclusion:**

There is potential to integrate U.S. federal climate policy and health policy, and doing so is perceived as advantageous by most. While there are barriers to climate-health policy integration and advancement, there are also promising opportunities, which may be more relevant under future presidential administrations or at the sub-federal level.

## Article highlights

• U.S. federal climate policy and health policy are not well integrated.
• Integration can build support for climate policy and maximize mutual benefits.
• Education, coordination, and advocacy are needed to advance climate-health policy.

## 1 Introduction

The health harms of climate change are numerous and far-reaching. For example, extreme heat exacerbates cardiovascular disease, kidney disease, mental health conditions, and pregnancy and childbirth risks [1]. These health harms disproportionately affect disadvantaged populations, such as low-wealth communities and communities of color [2,3]. Additionally, climate disasters impede healthcare services while increasing demand for these services [4,5].

Equally, addressing climate change would greatly benefit public health. Cutting fossil fuel emissions could save hundreds of thousands of lives in the U.S. through air quality benefits alone [6]. Climate solutions would also improve health through increased physical activity and healthier diets [7,8], and these improvements would reduce healthcare costs [9]. Growing recognition of these crucial intersections between climate and health solutions have spawned new interdisciplinary frameworks linking these topics, such as One Health and Planetary Health [10,11].

In the U.S., several federal policy initiatives have begun to address climate issues together with health issues. Examples include the Centers for Disease Control and Prevention’s Climate and Health Program [12], the Department of Health and Human Services’ Office of Climate Change and Health Equity [13] and voluntary Health Sector Pledge [14], the National Integrated Heat Health Information System [15], and the National Institutes of Health’s Climate Change Initiative [16]. Nevertheless, federal climate policies and health policies remain mostly separate [17,18]. Furthermore, the current president is unsupportive of both climate policies and public health policies [19], let alone the intersection of the two, and is cutting funding and staff in both areas across the federal government [20].

While many stakeholders have called for greater climate-health policy integration [4,21,22], barriers and opportunities for integration remain underexplored. This study examines U.S. federal policy stakeholders’ views of the current status of climate-health policy integration; their vision for whether and how climate policy and health policy should be integrated; and key barriers, opportunities, and strategies. It is part of a multinational study, with similar research conducted in Brazil, Caribbean nations, Germany, India, Kenya, and the U.K․ [23]. Our research focus and analysis of policy opportunities are informed by Kingdon’s multiple-streams model of policymaking, which distinguishes between problems (how issues are conceptualized as such and become salient to policymakers), policies (the development of proposals), and politics (shifts in power, public opinion, and the influence of interest groups) [24]. We note that this research was conducted prior to Political Administration; while this likely renders many of the recommendations unfeasible in the short term, they may be actionable under future political circumstances, at different scales (such as the state or local level), or in different nations/regions.

## 2 Materials and methods

From January 11th, 2024 – April 24th, 2024, we interviewed 65 experts working on climate policy (*n* = 20), health policy (*n* = 12), the climate-health intersection (*n* = 26), and related areas (*n* = 7) such as transportation, agriculture, and housing. We identified potential participants through personal contacts, online searches, and snowball sampling, and recruited them through individualized email invitations. Participants included congressional staff (*n* = 8), executive branch and federal agency employees (*n* = 33), policy experts at academic institutions (*n* = 4) and think tanks (*n* = 6), and policy advocates (*n* = 14). The sample included individuals with a range of career stages. Interviews were conducted via Zoom, ran between 11 and 55 minutes, and were auto-transcribed. Two researchers (JF and JE) conducted a qualitative content analysis of the transcripts in ATLAS.ti using a combination of deductive and inductive coding. Deductive codes were drawn from the interview question topics, and inductive codes were generated based on themes emerging from participants’ answers.

While this approach enabled us to identify common themes across the participants’ responses, it is important to note that the sample of participants is not representative of U.S. policy stakeholders, and is also based on the researchers’ subjective interpretations of their statements. The findings provide valuable insights on the participants’ perspectives of climate and health policy, but should not be interpreted as representing the views of the broader policy community. To avoid giving the false impression that our findings are statistically representative, we generally describe the results using phrases such as “many participants” rather than exact numbers. The researchers also note their positionality as individuals concerned about climate change and personally supportive of policy action at the intersection of climate and health. In order to limit the influence of these biases, we ensured that we asked our interview questions in neutral ways (e.g., “Should climate policies and health policies be more closely linked than they currently are, or more separated?”) and grounded our findings strongly in the participants’ statements.

The analysis was informed by feedback from some research participants, as well as policy stakeholders who participated in a briefing on our preliminary findings. The research was approved by the George Mason University Institutional Review Board (12/14/2023, reference number FP00001847) and was conducted in compliance with all applicable laws and institutional guidelines, including observing privacy rights and obtaining informed consent. Further methodological details are available in the supplementary materials.

## 3 Results

### 3.1 Current status of integration of climate policy and health policy

Consistent with prior research [17,18], most participants perceived that climate-related and health-related research and policymaking remain mostly separated at the national level. Examples include a lack of interaction between climate stakeholders and health stakeholders in congressional offices and the lack of funding for healthcare resilience in the Inflation Reduction Act. Participants observed, however, that climate policy and health policy have become more integrated in recent years. They attributed this change to the efforts of climate-health advocates, a growing understanding of the links between climate, racial justice, health, and other issues, and the actions of the Biden administration. In particular, they highlighted interagency working groups formed under the Biden administration, such as the Interagency Working Group on Extreme Heat. Multiple federal employees also noted the recent establishment of climate-health programs in the Department of Health and Human Services (HHS) and other agencies, characterizing these initiatives as promising, but still in an early phase. Participants additionally noticed that climate-related and health-related policy and programs were more frequently intertwined in certain domains, such as air quality, disaster response, and extreme weather.

### 3.2 Views about optimal integration

Almost all participants felt that climate policy and health policy should be more integrated. They commented that policy integration seemed sensible given the inherent interrelatedness of the issues, and believed that integration would help ensure that climate was adequately considered in health policy and vice versa, maximize the health benefits of climate policy and the climate benefits of health policy, and enhance support for climate policies by linking them to personally relevant health concerns. A commonly mentioned goal was making healthcare systems more resilient to climate change and lowering their carbon emissions. Participants also thought climate policy and health policy could be beneficially linked in work on public health, air quality, agriculture, food and water, transportation, housing, and urban development.

Some conservative participants believed that it would be better to keep climate policies and health policies separate. One participant was concerned that joint climate-health policies might not adequately advance either climate or health goals. Another participant warned that introducing climate policy into health policy could alienate conservatives, some of whom feel that climate issues are being introduced into policy contexts where they do not belong (e.g., the decarbonization of healthcare systems). However, this participant was in favor of integrating climate policy with health policy in the context of air pollution because they saw this as a case in which fossil fuel emissions are clearly and directly linked to health harms.

### 3.3 Barriers to optimal integration

**Funding.** Lack of funding was a commonly mentioned barrier to climate-health policy integration and advancement. Participants especially commented on the lack of funding for federal agency initiatives, including both interagency working groups and intra-agency bodies such as the Office of Climate Change and Health Equity.

**Conservative opposition.** Several participants traced the lack of federal funding to conservative congressmembers’ reluctance to fund programs related to climate change or health equity. They also commented on conservative presidential administrations’ disruptions of climate initiatives. Some participants attributed conservative opposition to the influence of the fossil fuel industry, but two conservative participants suggested that it was due to a perception of climate policy as a vehicle for addressing other progressive causes intersecting with climate action (e.g., the Green New Deal).

**Low awareness and understanding.** Low public awareness of the health harms associated with climate change was another commonly mentioned barrier. Participants added that climate experts, health experts, and congressional policymakers were often insufficiently informed about climate-health links. One congressional staffer voiced surprise at how little they heard about the climate-health intersection; another participant hypothesized that policymakers might not be aware of the depth of their constituents’ concern about these issues.

**Data for decision-making.** Participants said there was a lack of data needed for climate-health policymaking. They reflected that it was challenging to quantify the health harms of climate change: harms may be underreported if those affected do not seek medical care, and there are difficulties surrounding the attribution of hospital visits and the tracking of associated economic costs. They also reported a need for more data on the health benefits and healthcare cost savings of climate solutions. One participant additionally mentioned that there are restrictions to sharing data, once obtained, from states to the federal government, which makes it challenging to compare the outcomes of initiatives (e.g., heat resilience initiatives) across localities.

**Silos.** Silos between federal agencies were also commonly mentioned as an impediment to climate-health policy integration. Collaboration between federal agencies sometimes occurred, but was challenging due to agencies’ separate funding streams and lack of technical interoperability (e.g., the use of different units of measurement across the energy system versus the health system). Some federal agency employees also felt constrained by their congressional mandate to only focus their resources on work directly related to that mandate. Interagency working groups, while generally successful, were considered time-consuming and difficult to balance with other work priorities, particularly when participation was voluntary. Likewise, participants pointed out silos between health and climate communities outside government. They observed that climate experts and health experts received different training, communicated differently, and had different priorities and viewpoints. Another source of division was that health professionals were reportedly concerned that advocating for climate policy could reduce the public’s trust in them. Other barriers to the participation of health professionals in climate work included the prioritization of other public health issues (e.g., cancer), and limited resources for both public health and healthcare.

**Disinformation, climate skepticism, and misperceptions.** In addition to these obstacles on the health side, participants identified many factors impeding climate policy. Examples include communities’ belief that their economies depend on fossil fuels; opposition to regulations and taxes; the perception of climate change as abstract and distant; and the view that climate action incurs local costs in exchange for global benefits. Regarding this last perception, participants commented that, in fact, the health benefits of climate action are often realized locally and in the near term. Participants also mentioned fossil fuel industrys’ misinformation campaigns and the resulting rise of climate skepticism. While the analogous rise of health misinformation was not directly mentioned, participants did note that public health policy had become more politically contentious following the COVID-19 pandemic, alluding to misinformation related to vaccination and mask-wearing.

**Time horizons in policymaking.** Some participants noticed a similar preoccupation with minimizing immediate costs among policymakers to the detriment of long-term planning. They ascribed this short-termism to the pressures of election cycles, which only reward policy successes that materialize in four-year windows. Another reported challenge for policymakers was the need to act swiftly, yet substantively involve impacted communities in decision-making, which could be time-consuming due to the need to build trusting relationships over multiple interactions.

**Weak policy support for local climate change mitigation.** Finally, a few participants said there was less support for addressing the indirect health harms of climate change, such as vector-borne disease, than the immediate health harms of fossil fuel pollution, such as high asthma rates in communities near highways. They also commented that communities might be less supportive of local climate solutions if they were mainly experiencing health harms from non-local pollution (e.g., air pollution from sources outside their control), since local climate solutions would not sufficiently alleviate those harms.

### 3.4 Opportunities to advance integration

**Growing public concern.** Several participants highlighted growing public awareness of the health harms of climate change, such as the air pollution resulting from wildfires. As one climate-health advocate commented, *“We dont have to do that much work anymore to connect the dots that climate change might lead to your kid having an asthma attack. Everyone in America breathed it last summer.”* Participants also observed increasing support for climate solutions, particularly among youth and conservatives, and increasing momentum for climate-health work among federal employees and at international climate negotiations. Furthermore, several participants themselves expressed interest in learning more about the climate-health intersection.

**Recent major legislation.** A commonly mentioned opportunity was the funding for climate-health initiatives included in the Inflation Reduction Act and Bipartisan Infrastructure Law, which participants saw as underused resources. For example, the IRA included provisions to incentivize solar panel installation and other similar measures, which were available to clinics and other healthcare facilities. Participants suggested educating communities about this funding and encouraging them to apply.

**Executive Actions.** In terms of opportunities to take action, one participant emphasized the President’s ability to declare a climate emergency and deny permits for fossil fuel exploration and infrastructure. However, some participants warned that executive actions would not have the staying power of congressional legislation, since future administrations could overturn them.

**The feasibility of smaller, more focused wins.** Due to conservative opposition, near-term opportunities for congressional legislation on the climate-health intersection were generally seen to be scarce, and participants felt it would be challenging to pass a comprehensive climate-health bill. Instead, they believed there might be opportunities to integrate climate-health objectives into diverse policy areas such as agriculture, transportation, and housing. A communications strategist commented, *“The next big climate legislation that people are thinking about is the Farm Bill and the ways to support farmers and more regenerative agriculture and land conservation policies—ways to protect water, protect our lands, protect food. I think those all have obviously a lot of connections to health.”*

**Bipartisan support for clean energy.** Participants also identified areas of climate policy that they thought could garner bipartisan support. Though not explicitly concerned with health, these policies would ultimately benefit health by alleviating climate change. These policy areas include renewable energy — especially clean energy permitting reform — and carbon border adjustments. They were primarily brought up by participants working in the climate sector, but also by two participants working at the climate-health intersection.

**The health economic benefits of climate action.** Finally, several participants pointed out that climate solutions present an economic opportunity through reduced health costs. One participant, an academic researcher, estimated that decarbonization would save thousands of lives and hundreds of billion dollars annually.

**Currently untapped data.** Participants also noted the opportunity to analyze existing climate-health data and collect further data through federal agencies such as the Center for Medicare and Medicaid Innovation.

### 3.5 Strategies to advance integration

#### 3.5.1 Communication strategies

**Framing climate change in terms of health.** Participants suggested that framing climate change in terms of health could help connect climate change to audiences’ immediate priorities. To accentuate this immediacy, they suggested focusing on specific, local, tangible, relevant, and present or near-future health harms of climate change. They believed that a health frame could appeal to conservative audiences — a view that aligns with the findings of experimental research on the subject [25,26]. Participants suggested focusing on both the health risks of climate change (such as asthma, mental health problems, and infectious disease) and the health benefits that could be enjoyed through climate solutions (such as lives saved and illnesses alleviated or avoided). They particularly recommended emphasizing how policies could be “win-wins” for both health and the climate. Several participants also suggested mentioning the economic facets of the climate-health intersection, such as the economic costs of health harms or the job opportunities related to climate solutions.

**Using health professionals as messengers.** In general, participants thought that health professionals could be effective messengers about the climate-health intersection because of their status as trusted experts. One participant commented that health organizations may themselves be most receptive to climate communication coming from other health organizations. A few participants were concerned, however, that certain audiences might no longer trust health authorities because of the political polarization and science denialism of the COVID-19 pandemic. Other recommended messengers include constituents (when addressing policymakers), frontline community members, and youth.

**Selecting target audiences.** In terms of advocacy to policymakers, recommendations for target audiences varied. Some participants suggested contacting policymakers who are already sympathetic to climate-health concerns, but one participant believed it was more strategic to try to persuade opposed policymakers. Another recommendation was to engage policymakers who are on congressional committees relevant to climate and/or health. No matter the audience, participants emphasized the need to build trusting relationships over time through “soft touches,” i.e., non-adversarial conversations not only centered on demands. In terms of public-facing communication, participants recommended engaging frontline communities, faith communities, parents, those who accept climate science but do not see climate change as an urgent priority, and constituents who can, in turn, advocate to legislators.

**Engaging conservative audiences.** Respondents said that in communication with conservative audiences, influential messengers include faith leaders and healthcare providers. They suggested asking questions and listening, highlighting aspects of climate policy that resonate with conservative stances, and imbuing messages with conservative values such as accountability, purity (e.g., cleanliness or pollution of water), reverence for life, and love for the land. They also cautioned against using a patronizing, shaming, or apocalyptic tone. However, participants had differing views on whether to avoid certain polarized terms such as “climate change,” “health equity,” and “environmental justice”: some recommended avoiding these phrases, while others were concerned about what could be lost in translation. One participant recommended using these polarized terms frequently in order to desensitize conservative audiences to them.

#### 3.5.2 Broader strategies

**Education.** Participants recommended incorporating a climate-health focus into curricula in high schools, universities, and businesses. They noted that climate experts should be taught more about health and vice versa. Recommended topics included raising awareness of the health impacts of climate change, debunking climate misinformation, and offering trainings on emergency preparedness. In addition to classroom education, participants recommended knowledge- and data-sharing across federal agencies and sectors.

**Research.** More research is needed to quantify the health harms of climate change and the health benefits of climate solutions, evaluate the outcomes of current initiatives, and examine the causes of opposition to climate policy. Participants recommended using participatory methods such as citizen science. Participants also identified a need for more integrated approaches to climate research and health research, such as a national climate-health research framework, which could potentially be coordinated through the U.S. Global Change Research Program’s Interagency Crosscutting Group on Climate Change and Human Health. Additionally, the findings suggest a need for more research to develop an ambitious yet feasible climate-health policy agenda; to pilot test proposals to integrate climate work with health work across federal agencies and professional communities; and to evaluate communication strategies, e.g., for engaging conservatives about the equity aspects of the climate-health nexus.

**Enhancing coordination.** Participants saw a need for more central coordination of federal climate-health work. While they noted that White House liaisons had been helpful in coordinating interagency climate-health working groups, they suggested that it would be best to have a federal office serve as a coordinator to reduce vulnerability to presidential administration changes. One participant recommended the Office of Climate Change and Health Equity for this coordinating role because its work already intersected with multiple agencies. Other (or complementary) options included increasing funding for interagency working groups, better supporting intra-agency programs and training (especially for career staff), and involving health professionals in climate policymaking.

**Focusing first on adaptation and incentives.** While there was no strong consensus on how to overcome congressional gridlock, a few participants suggested focusing on adaptation, and the associated costs, as a way to build support for mitigation. Two participants mentioned that, in the absence of a legislative path for regulations to curtail fossil fuel pollution, it may be strategic to focus first on passing incentives such as those in the Inflation Reduction Act. Others recommended policymaking strategies included using participatory frameworks and, as mentioned previously, integrating climate-health policy into other, related legislation.

**Harnessing existing financial resources and cultivating new ones.** To free up resources for climate-health initiatives, participants suggested not only making the most of existing funding opportunities, but also raising additional funds through innovative financing approaches. For example, one participant suggested that climate adaptation could be funded in part through Medicare.

**Electing and cultivating policy champions.** Several participants remarked on the importance of climate-health policy champions in Congress, the White House, federal agencies, and outside government. They suggested voting in new climate-health champions as well as cultivating interest among existing policymakers through events such as congressional hearings. They also recommended placing climate-health champions as staffers in congressional offices, through university or non-profit funding, and including them in National Academies advisory committees.

**Persistent advocacy.** Participants explained that these changes in leadership, policymaking, and implementation ran counter to entrenched power structures, such as the influence of money in politics, that could be overcome only with sustained advocacy. They recommended grassroots organizing, coalition-building, protest, social media activism, attracting media interest through citizen science, and pressuring the fossil fuel industry. One suggestion for how to pressure the fossil fuel industry was to revoke its social license and instead popularize renewable energy. Participants emphasized that activists and advocates need to be persistent, avoid making concessions prematurely, and refuse false choices between the health of humans and the health of the climate.

Table 1 summarizes the proposed strategies in relation to the barriers discussed earlier:

**Table 1:** Barriers and proposed strategies to overcome them.

Barrier	Proposed strategies
Lack of funding	• Leverage existing funding opportunities • Develop innovative funding mechanisms
Conservative opposition	• Elect and cultivate climate champions • Build strong progressive coalitions that can overcome conservative opposition • Tailor communication to conservative values • Pursue policies that have bipartisan support • Pass components of policies piecemeal • Persuade opponents to be less vocal
Limited awareness	• Educate policymakers, climate experts, health experts (including clinicians), and the public about climate-health links • Include the climate-health nexus in curricula • Engage the public in citizen science • Increase media coverage • Hold congressional hearings
Lack of data	• Conduct additional research on: health harms of climate change, health benefits of climate solutions, economic ramifications, program outcomes, communication techniques, root causes of opposition, and strategic policy roadmaps • Establish a national research framework • Share existing data (as permitted) across government agencies and with the public • Incentivize a systems approach to science
Silos between climate stakeholders and health stakeholders (both in government and in the private sector)	• Create and strengthen interagency working groups • Establish central coordination • Develop tools to facilitate technical interoperability • Institutionalize climate-health programs in each relevant agency • Embed health stakeholders in climate policymaking • Hire climate-health experts
General health sector challenges	• Increase funding and capacity for public health and healthcare, both in the U.S. and abroad • Improve advertising of existing funding opportunities through the IRA • Support healthcare providers to track climate impacts and increase climate resilience • Identify benefits of climate solutions for healthcare
General climate policy challenges	• Conduct persistent activism and advocacy to reduce the influence of the fossil fuel industry, counter its misinformation, and champion solutions • Frame climate in terms of health to make it more relevant to audiences’ immediate concerns • If there is not a viable path for mitigation policies, focus first on adaptation
Low policy support for climate mitigation (e.g., due to short-term pressures)	• Make more salient the local, short-term health benefits of climate solutions • Pursue policies that result in cost savings • Emphasize health, environmental, and economic “win-wins”

## 4 Discussion

These results resonate with previous findings that national climate policy and health policy are not well integrated in the U.S. context [15,16], yet add some promising insights: (1) stakeholders working on climate and/or health policy generally believed it would be beneficial to integrate these two areas, (2) they perceived growing momentum towards climate-health solutions, and (3) they saw a wide array of opportunities to build on this momentum. Participants identified a variety of positive developments that were advancing climate-health policies, such as inter-agency working groups, the Office of Climate Change and Health Equity, and sustained advocacy initiatives that leverage public trust in health professionals. The flourishing of these initiatives under the Biden administration, and their subsequent reversal under the current administration, underscore the strong influence of executive leadership. To continue this work, it is important that climate and health policy stakeholders find ways to maintain collaboration and dialogue across disciplines and sectors, such as cultivating funding sources beyond the federal government.

Examples of opportunities include reducing healthcare costs through climate solutions and introducing climate-health legislation into relevant policies in areas such as agriculture, transportation, emergency response, and housing and urban development. Participants also shared many strategies for making progress towards climate-health solutions, such as educating policymakers and the public, cultivating and empowering policy champions, and increasing interaction between climate experts and health experts both within and outside government.

Nevertheless, climate-health policy faces significant obstacles. One of the most prominent challenges mentioned was Congressional gridlock. Relevant recommendations include organizing voters to elect climate-health champions, countering political polarization through education and persuasive communication, and integrating pieces of climate-health legislation into other bills where possible. Another notable challenge was how to build bipartisan support for climate-health policy while prioritizing equity and justice. Given some conservatives’ aversion to overt discussion of equity and justice, it may be necessary to further explore ways of engaging conservatives on these topics. For instance, future research could test the proposed strategy of desensitizing conservative audiences to explicitly justice-focused messages through repetition against the strategy of rephrasing such messages in less polarized terms.

Another increasingly relevant challenge is mis- and distrust of medical professionals. Compared to before the COVID-19 pandemic, trust in physicians, hospitals, medical scientists, and public health officials has declined [[27], [28], [29]]. This trend may weaken the potential depolarizing effect of health frames on climate change discourse. On the other hand, perhaps health professionals will be less reluctant to engage in climate advocacy if their credibility is already being attacked on other grounds (e.g., through a “nothing-to-lose” mindset). Encouragingly, previous research has demonstrated that engaging in climate advocacy actually increases health professionals’ credibility [30].

More broadly, substantial uncertainties remain around what is the most impactful and feasible climate-health policy agenda, particularly in the volatile partisan environment of U.S. politics and the recent shift towards an administration opposed to climate-health initiatives. Federal funding challenges and political barriers are likely to intensify, underscoring the importance of seeking alternative funding sources and strengthening communication, education, and advocacy. State and local policymaking is likely to take on increased importance, suggesting a pressing need for research that explores these avenues (which was beyond the scope of the current study). For example, prior research has shown that state and local climate-health initiatives suffer from a lack of adaptation capacity and would benefit from federal resources [31,32]. With diminished federal funding opportunities, it is essential to identify avenues for enabling state and local adaptation work to continue. Further research is also needed to understand how best to build a more diverse coalition of support for climate-health policy across the United States, especially among frontline communities and conservatives, and engage supporters in voting, advocacy, and activism.

Furthermore, future research could build on our findings by field testing and refining participants’ recommended strategies, including through the use of other types of methodologies and with larger sample sizes. Research on other national contexts could be illuminating, especially those with characteristics distinct from the U.S. (e.g., Global South countries and countries with less political polarization). There is also a need for further in-depth research into policymaking opportunities and barriers around specific types of climate-health policies, such as climate adaptation, sustainability of health systems, and disaster preparedness, as well as further research on the integration of a climate-health focus into related policy domains, such as agriculture and transport.

Our study has several limitations. As is often the case when recruiting policy elites, we were not able to gain access to everyone we wished to interview. Those who agreed to participate may have been more supportive of climate-health policy than those who declined, resulting in a sampling bias. Additionally, our study scope allowed us to explore participants’ views, but not to assess the validity or accuracy of their statements, or which of their suggested strategies are most viable. To mitigate the possibility of misunderstandings or inaccuracies, we sought feedback from expert stakeholders on our draft report. Please see the methods section for more limitations related to the qualitative methodology.

## 5 Conclusion

This study sought to understand U.S. stakeholders’ views on whether and how to integrate climate policy and health policy. We found that most participants were in favor of integration, and many observed signs of progress, including growing awareness; emerging funding, data, and technologies; and new federal programs within and across agencies. However, they identified many challenges, such as political polarization, lack of resources, and silos between federal agencies and between professional communities. To address these challenges, they recommended a wide range of strategies, including education, research, relationship-building, additional federal program development, advocacy, and activism. These strategies could make important strides toward policies to protect the climate and human health.

## Data statement

Due to the sensitive nature of some of our interview questions, the data collected for the study are confidential and will not be archived publicly.

## CRediT authorship contribution statement

**Julia Fine:** Writing – review & editing, Writing – original draft, Methodology, Investigation, Formal analysis, Data curation. **Joshua Ettinger:** Writing – review & editing, Writing – original draft, Methodology, Investigation, Formal analysis, Data curation. **John Kotcher:** Writing – review & editing, Project administration, Methodology, Funding acquisition, Conceptualization. **Matto Mildenberger:** Writing – review & editing, Methodology, Conceptualization. **Anthony Leiserowitz:** Writing – review & editing, Conceptualization. **Edward Maibach:** Writing – review & editing, Resources, Project administration, Methodology, Funding acquisition, Conceptualization.

## Declaration of competing interest

Three of the authors are Guest Editors for this journal and were not involved in the editorial review or the decision to publish this article.

The authors declare the following financial interests/personal relationships which may be considered as potential competing interests:

JE reports an honorarium from the Columbia University Mailman School of Public Health.

JK reports funding from WHO; consulting fees from Pro-Change Behavior Solutions; and an honorarium from the Space Science Institute.

EM reports grants from the Robert Wood Johnson Foundation, the Energy Foundation, and the Kresge Foundation; an honorarium for teaching at the University of Colorado; support from the Climate and Health Foundation to attend New York City Climate Week; and his role as a board member of the Global Climate and Health Alliance.

## Appendix Supplementary materials

Image, application 1
