# Supplementary material for: Advancing and integrating climate and health policy in the United States: Insights from national policy stakeholders
Source: J Clim Chang Health. 2025 Sep 4;25:100485. doi: 10.1016/j.joclim.2025.100485 (PMC12851067; doi:10.1016/j.joclim.2025.100485)
Supplement: Supplementary file 1 [file mmc1.docx]

# **Supplementary information on methodology**

## **Recruitment**

In order to create a purposive sample of interview participants central to the health and climate policymaking ecosystem, we recruited individuals who met both of the following criteria:

1. Currently working or has recently worked on federal climate policies, health policies, or climate and health policies in the United States. This includes work on policy implementation.
2. Currently working or has recently worked in academia; as a policy advocate; for the United States federal government; as legislative staff in the United States Congress; or at a think tank.

We also recruited individuals working on policies strongly relevant to climate and health such as agriculture and transportation.

We recruited potential participants by sending email invitations to personal contacts of the research team; through snowball sampling with participants (i.e., asking participants for recommendations of other individuals we could potentially interview); and by identifying individuals with relevant expertise through Google search and on Linkedin.com.

We conducted a total of 65 semi-structured interviews during the period of January 2024 to April 2024. Though our sample was non-representative, we recruited participants across a range of sectors (Figure 1), areas of expertise (Figure 2), and political beliefs (Figure 1).

## **Interviews**

The interviews were semi-structured insofar that we created a pre-established list of interview questions, but maintained flexibility to ask additional questions and to ask participants to further elaborate their responses. The list of prepared questions for interviewees working on climate policy is available below; these questions were adjusted for interviewees working on health policy, both climate and health policy, and related policy areas. The questions were divided into categories based on our research questions. They sought to elicit participants’ views and experiences related to the current national status of climate and health policies; ideals for the climate and health policy relationship; barriers to advancing and integrating climate and health; and opportunities and strategies for overcoming barriers.

1. How much do human health impacts or benefits feature in your work?
   1. Could you briefly give me an example of a recent time when health issues came up in your work on climate policies?/Can you think of a time in the past when health issues came up in your work on climate policies?
      1. What kinds of people or organizations were involved, and what positions were they advocating?
      2. What were the outcomes?
      3. How typical was this example of how health issues generally come up in your work? What about it was typical or atypical?
2. To what extent are health and climate policies linked, or not, in [their country/region]?
   1. *(If links are mentioned)* In what ways are they linked?
3. Should they be more closely linked than they currently are in [their country/region], or more separated?
   1. *(If they think climate and health policy should be linked in any way)* What are the benefits of linking climate and health policy?
   2. Do you think incorporating health considerations can build support for climate policies? Why or why not?
4. How could climate policy or policymaking procedures be improved in [their country/region] to more fully incorporate health considerations?
   1. *(If they don’t mention specific policies or policymaking procedures)* Are there specific climate policies or policymaking procedures that could be created, improved, or removed to more fully incorporate health considerations?
   2. Do any specific success stories come to mind?
   3. Are there models from other countries that you have used, or are considering using, in your work?
5. What do you think is the biggest barrier to achieving the types of policy changes that you described?
   1. What would it take to overcome this barrier? *(such as communication approaches, resources, and other strategies)*
   2. Are there any other important barriers? *(If so, ask how to overcome them)*
6. What opportunities do you see to achieve the types of policy changes that you described earlier in our conversation?
   1. Are there any other opportunities you see?
   2. What would it take to move forward with these opportunities?
7. *(If relevant to participant’s background)* In your experience, what (if anything) has worked to influence policymakers to support climate policies?
8. *(If relevant to participant’s background)* How, if at all, have health considerations influenced your country’s positions in international climate negotiations? (such as COP28, for example)
9. Is there anything else that didn’t come up in our conversation that you want to share before we end?

The interviews ranged from 11 to 55 minutes, with an average time of 34 minutes. They were conducted over Zoom by either one or two members of the research team. The interviews were recorded and automatically transcribed using Zoom, with subsequent manual cleaning up of transcripts to correct occasional transcription errors, organize the text into a more readable format, and anonymize identifiable information.

To obtain informed consent, participants were sent a participant information sheet prior to their interviews. After joining on Zoom and before starting the interview, they were asked to verbally approve the participant information sheet. They were given the option to use video and audio, or just audio, depending on their preference. They were also asked to provide their preferred anonymous identifier for the report (e.g., “congressional staffer working on climate policy”).

## **Analysis**

The transcripts were coded in ATLAS.ti using mixed deductive and inductive manual qualitative content analysis (Fereday & Muir-Cochrane, 2006). The six deductive codes were created based on the interview questions prior to starting the coding process:

1. Experiences related to climate/health policy [participant describes how climate and/or health comes up in their work, whether on a daily basis or over a longer period of time]
2. National status of climate/health policy [participant describes the current relationship of climate and health policy at a national level in their country]
3. Ideals for climate/health policy [participant describes what the relationship between climate and health policy should look like, in their opinion]
4. Barriers to climate/health policy [participant describes challenges for advancing climate and health policies]
5. Opportunities for climate/health policy [participant describes promising ways to advance climate and health policies]
6. Strategies for climate/health research, policymaking, and implementation [participant describes methods for advancing climate and health policies, such as communication strategies, political strategies, and any other approaches]
7. Inductive codes within these deductive categories were created during the coding process.
8. Two members of the research team conducted the coding. We coded three transcripts independently and then discussed any differences after each one to help ensure a similar coding approach. We then separately coded the full corpus of transcripts with regular meetings to discuss and iteratively update the codebook.
9. After the transcripts were fully coded, we used a list of pre-established analysis queries to guide the analysis of the coded data. The list of queries is available below.
10. Experiences
    1. To what extent, and in what ways, do health experts experience links between climate change and their work? (and vice versa)
    2. How often, in what contexts, and in what ways do climate and health experts interact? In what contexts are they siloed?
11. Current national situation
    1. To what extent, and in what ways, do participants feel that climate and health are linked or separate in national policymaking?
    2. To what extent and in what ways did participants perceive that health considerations were influencing their nation’s stance in international climate negotiations?
12. Ideals
    1. To what extent, and in what ways, do participants feel that climate and health policymaking should be more integrated or separate in their country?
       1. Integrated or separate in which respects?
          1. Behind-the-scenes policy planning
          2. Policy instruments
          3. How policies are communicated about
13. What success stories or models did participants mention, if any?
    1. Where did these success stories come from?
    2. How did they recommend building on these successes?
14. Barriers
    1. What are the reported barriers to climate and health integration?
    2. Which barriers were most commonly mentioned, and by whom?
    3. Which were rarely mentioned, but potentially important?
    4. What are the reported barriers to climate and/or health policy advancement (not necessarily integrated)?
    5. Which barriers were most commonly mentioned?
    6. Which were rarely mentioned, but potentially important?
15. Opportunities
    1. What are the reported opportunities (i.e., existing and/or developing conditions in the policy environment) for climate and health integration?
    2. Which opportunities were most commonly mentioned?
    3. Which were less commonly mentioned but particularly compelling?
    4. What are the reported opportunities (i.e., existing and/or developing conditions in the policy environment) for advancing climate and/or health policy (not necessarily integrated)?
    5. Which opportunities were most commonly mentioned?
    6. Which were less commonly mentioned but particularly compelling?
16. Strategies
    1. What strategies and tactics did participants suggest for advancing climate and health policymaking?
       1. Communication strategies/tactics for engaging policymakers
       2. Communication strategies/tactics for engaging the public
       3. Non–communications strategies
    2. To what extent, and in what ways, did participants feel that a health frame could increase or decrease support for climate policy?
    3. To what extent, and in what ways, did participants feel that a climate frame could increase or decrease support for health policy?
17. Other relevant issues
    1. To what extent, and in what ways, does climate justice/equity come up in the participants’ responses?
    2. To what extent, and in what ways, do economic impacts and benefits come up in the participants’ responses?
    3. What kinds of climate impacts were discussed?
    4. Which specific policies were discussed?
18. Overall synthesis
    1. Distribution of knowledge
    2. Which climate and health policy topics did interviewees know the most about?
    3. Which climate and health policy topics did interviewees know the least about?
    4. Policymaking themes
    5. Were there any themes across the participants’ experiences of policymaking processes not covered by the above questions? For instance, what were some of the positive/rewarding aspects and what kinds of frustrations/difficulties did they experience?
    6. Good quotes
    7. Were there any statements or stories that particularly stuck out? What makes these instances unique and important?

## **Engagement with Global Climate and Health Alliance stakeholders**

We solicited feedback on our research questions via a survey administered to stakeholders on the mailing list of the Global Climate and Health Alliance in November 2023 (n = 264). We invited these stakeholders, along with our research participants, to a briefing on our preliminary results in July 2024. We specifically asked them the following discussion questions:

1. How do these findings align or conflict with your experiences?
2. Are we missing any crucial insights or perspectives?
3. Is there one main takeaway that you see as most important? What is it and why?

We integrated their feedback, then solicited their feedback on our draft report via an online form in September 2024.
